# Cost analysis of Human Papillomavirus-related cervical diseases and genital warts in Swaziland

**DOI:** 10.1371/journal.pone.0177762

**Published:** 2017-05-22

**Authors:** Themba G. Ginindza, Benn Sartorius, Xolisile Dlamini, Ellinor Östensson

**Affiliations:** 1 Discipline of Public Health, School of Nursing and Public Health, University of KwaZulu-Natal, Durban, South Africa; 2 Epidemiology Unit, Ministry of Health, Mbabane, Swaziland; 3 Department of Medical Epidemiology and Biostatistics, Karolinska Institutet, Stockholm, Sweden; 4 Department of Women's and Children's Health, Karolinska Institutet, Stockholm, Sweden; Universidade Estadual de Maringa, BRAZIL

## Abstract

**Background:**

Human papillomavirus (HPV) has proven to be the cause of several severe clinical conditions on the cervix, vulva, vagina, anus, oropharynx and penis. Several studies have assessed the costs of cervical lesions, cervical cancer (CC), and genital warts. However, few have been done in Africa and none in Swaziland. Cost analysis is critical in providing useful information for economic evaluations to guide policymakers concerned with the allocation of resources in order to reduce the disease burden.

**Materials and methods:**

A prevalence-based cost of illness (COI) methodology was used to investigate the economic burden of HPV-related diseases. We used a top-down approach for the cost associated with hospital care and a bottom-up approach to estimate the cost associated with outpatient and primary care. The current study was conducted from a provider perspective since the state bears the majority of the costs of screening and treatment in Swaziland. All identifiable direct medical costs were considered for cervical lesions, cervical cancer and genital warts, which were primary diagnoses during 2015. A mix of bottom up micro-costing ingredients approach and top-down approaches was used to collect data on costs. All costs were computed at the price level of 2015 and converted to dollars ($).

**Results:**

The total annual estimated direct medical cost associated with screening, managing and treating cervical lesions, CC and genital warts in Swaziland was $16 million. The largest cost in the analysis was estimated for treatment of high-grade cervical lesions and cervical cancer representing 80% of the total cost ($12.6 million). Costs for screening only represented 5% of the total cost ($0.9 million). Treatment of genital warts represented 6% of the total cost ($1million).

**Conclusion:**

According to the cost estimations in this study, the economic burden of HPV-related cervical diseases and genital warts represents a major public health issue in Swaziland. Prevention of HPV infection with a national HPV immunization programme for pre-adolescent girls would prevent the majority of CC related deaths and associated costs.

## Introduction

Human papilloma virus (HPV) infection and specifically oncogenic HPV types 16 and 18 are highly associated with cervical abnormalities and invasive cervical cancer and other diseases on the cervix, vulva, vagina, anus, oropharynx and penis, all of which impact on people´s health and resource consumption in society [1]. According to the published literature, the cost for screening, management and treating patients with HPV-related diseases is related to the high health care costs and is suggested to be greater than the combined cost for the prevention and treatment of most prevalent sexually transmitted diseases (STIs) [2].

While screening methods advance and HPV vaccination introduction efforts gain momentum, information on the current evidence on the effectiveness of prevention methods is essential to reinforce further development and expansion of programmes [3]. Currently, the common prophylactic vaccines for cervical cancer target most prevalent oncogenic types of HPV 16 and 18, with nearly 100% type-specific protection [3, 4]. Two HPV vaccines have been available since 2006, the quadrivalent HPV vaccine (HPV-4) (Gardasil, Merck) [5] and the bivalent HPV vaccine (HPV-2) (Cervarix with AS04, GlaxoSmithKline) [4, 6]. The HPV-4 vaccine also targets non-oncogenic HPV types 6 and 11, which are the cause of genital warts [5]. Studies have demonstrated that both vaccines have more than 70% efficacy against the incidence of high-grade squamous intraepithelial lesion (HSIL), if administered to previously unexposed women [5–11]. Recently, nonavalent vaccine (HPV-9) targeting seven HPV types (HPV16/18/31/33/45/52/58) that contribute to 90% of cervical cancer cases plus HPV-6/11 has been licensed and recommended, based on evidence of vaccine efficacy and immunogenicity [12, 13]. HPV vaccination is now considered to be the primary form of cervical cancer prevention [4, 14].

In most health economic studies related to cervical cancer prevention in low-income countries (LIC), data have been reported as part of the bigger picture of the cost-effectiveness analyses [15–19]. Other costing research done in the LIC reported information on cervical cancer screening and diagnosis, but there are very few reports on treatment for cervical intraepithelial neoplasia (CIN), invasive cervical cancer (ICC) [20–22] and genital warts [23]. The estimated societal cost of non-carcinogenic HPV disease such as genital warts is also essential to establish the health sector budget since 32 million people are affected by genital warts annually and 90% of these are related to HPV6/11 [24]. Most of the cost analysis on HPV-related diseases have been done in the high-income and, middle-income countries and very few in the low-income countries, especially in Africa [25–29]. Such studies are critical in providing useful information for policymakers in low-resource countries concerned with the allocation of scarce resources to reduce the burden from HPV among the whole population [20]. However, to date there are no published studies for the Kingdom of Swaziland. To invest in public interventions for HPV immunization, it is essential to know the human and economic burden of HPV-related conditions. Estimates of the economic burden of a disease for a given period are useful for understanding the relative economic magnitude of a health problem [30]. The principal component of health economic analyses is cost analysis and it has direct implications for the cost-effectiveness analyses used for decisions of resource allocation for public health interventions [15, 31]. Therefore, the economic burden of HPV-related conditions needs to be further investigated and the present study is the first in Swaziland to estimate the cost of HPV-related conditions such as cervical lesions, cervical cancer and genital warts.

## Materials and methods

### Study area

**Fig 1:**
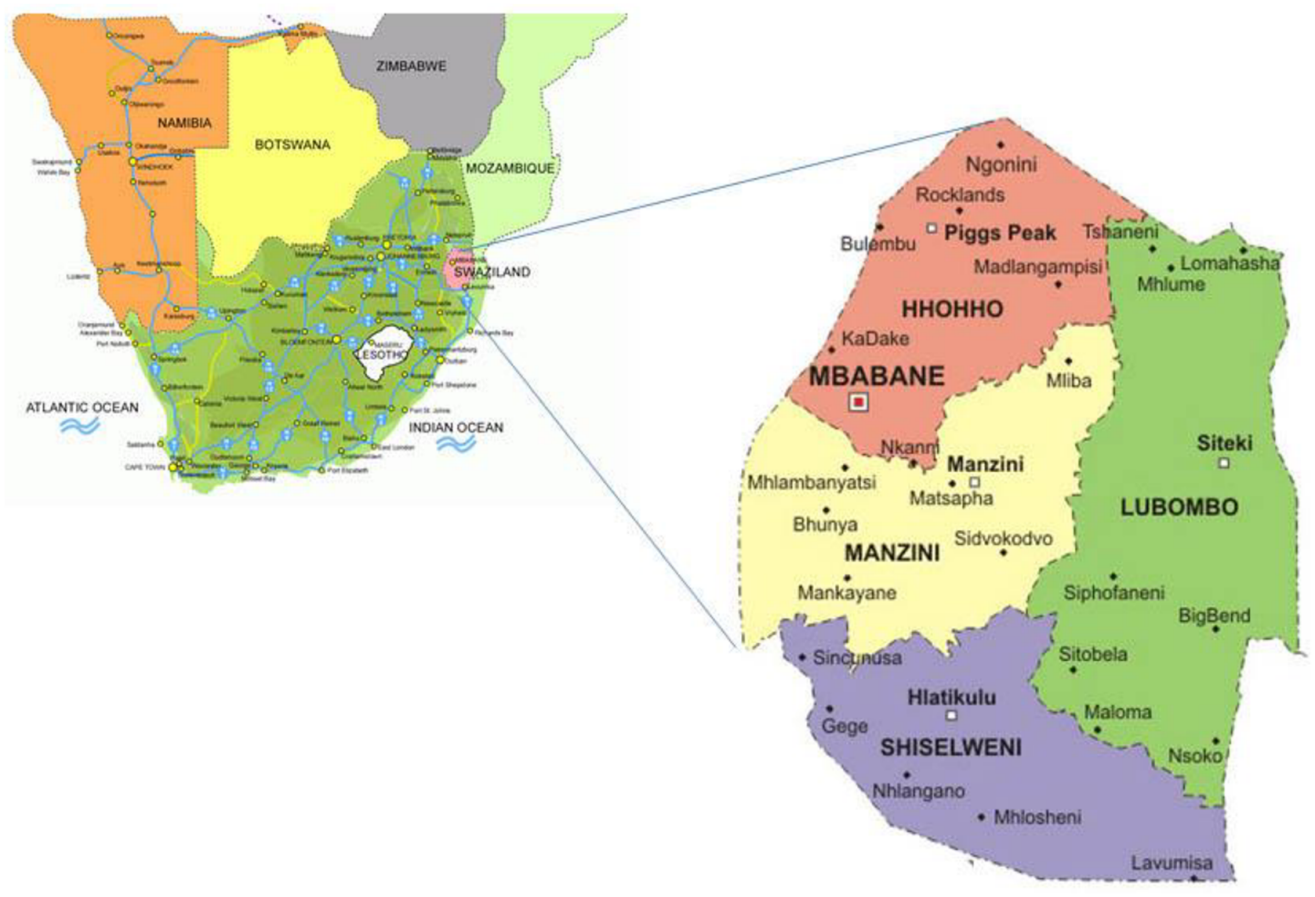
Map showing the location and political regions of Swaziland. **Source**: Macmillan. Geography and map of Swaziland Matsapha2005 [accessed 2012]. Available from: http://geography.about.com/library/blcswaziland.htm.

The Kingdom of Swaziland is a small, independent, low-resource nation in Southern Africa. It covers an area of 17,364 km ^2^ and is landlocked, with Mozambique to its east and South Africa to its north, west, and south [32, 33] It is divided into four political regions (Hhohho, Manzini, Shiselweni and Lubombo) (Fig 1) [33]. The 2007 population and housing census estimated the population of Swaziland to be 1 018 449, with 537 021 females, including a figure of 126 616 aged between 30 and 59 years [34]. Swaziland's currency is pegged to the South African rand, effectively relinquishing Swaziland's monetary policy to South Africa [35]. The government is heavily dependent on customs duties from the Southern African Customs Union (SACU), and worker remittances from South Africa supplement domestically earned income [35]. Swaziland’s GDP per capita makes it a lower middle-income country, but its income distribution is highly skewed, with an estimated 20% of the population controlling 80% of the nation’s wealth [35]. The GDP per Capita in Swaziland is equivalent to 32 percent of the world's average. GDP per capita in Swaziland averaged $2598.20 from 1970 until 2015, reaching an all-time high of 4057.28 USD in 2015 [35, 36]. As of 2014, more than one-quarter of the adult population was infected by HIV/AIDS; Swaziland has the world’s highest HIV prevalence rate [35, 37]. Additionally, the burden of high-risk HPV (hr-HPV) infection is high (46.2%), corresponding to 174,046 women (aged between 15 and 49 years old) in the country [38]. Currently, HPV testing is not part of both screening and management strategies. In addition, HPV immunization programme is not available in Swaziland. Furthermore, cancer patients are referred to South Africa for cancer staging and cancer treatment (chemotherapy, radiotherapy and brachytherapy) because such services are not available in Swaziland.

### Cost-of-illness

From the provider perspective we performed a cost-of-illness study which is a commonly used methodology to investigate the economic burden of diseases [39]. It´s a descriptive type of analysis estimating specific disease related costs and gives information on where these costs occur in a society. In addition, cost-of illness studies gives important reference data for economic evaluations for further guidance on how to allocate resources to improve resource efficiency. Economical evaluations are important for health policy makers when planning decisions regarding budget. An example could be the estimated cost a given country spends per year on caring for patients with cervical cancer. With an increased prevalence of HPV, the number of cervical cancer patients will increase and it´s important to plan for increased costs and interventions to reduce disease burden. Cost-of-illness studies are performed by using either the incidence or prevalence basis. Incidence-based approaches are usually used to estimate the life-time costs for a patient, from diagnosis to cure or death useful to estimate the effect of a specific treatment on future costs. The prevalence-based method considers all costs for a patient population in a geographical area for a given time period. The prevalence-based method has been previously used in for Sweden investigating costs for HPV-related cervical diseases and genital warts [40]. From this methodology, we used a top-down approach for the costs associated with health care performed at the hospital and a bottom-up approach to determine costs associated with primary care. Our study population were females diagnosed with cervical cancer, cervical lesions and genital warts.

### Cost data

We investigated only direct medical costs, which were divided into recurrent costs and capital costs [18]. Recurrent costs included personnel, travel, consumables/supplies (medical and non-medical), administration, utilities and overheads, while capital costs made up of equipment, buildings, vehicles and any other cost item with a useful life of more than one year and with an equivalent value of $50. All costs were computed at the 2015 price level and converted to dollars ($). All costs for screening and management of high-grade cervical lesions (HSIL) and genital warts were based on sources presented in Table 1.

**Table 1:** Data variables and source for costs regarding screening, management and treatment of cervical lesions, cervical cancer and genital warts.

Data /Parameter	Source	Prices source
**Estimated numbers of cervical cancer cases**		
Total number of cervical cancer 220 cases	Swaziland National Cancer Registry (SNCR), Ministry of Health), Report On Cases Of Cancers In Swaziland (2014–2015)	
**Screening**		
VIA screened	Monitoring &Evaluation, Strategic Information Department, Ministry of Health: Annual National Sexual and Reproductive Health programme report, 2015, Pages 15–16 Health Management Information System (HMIS)	Market price
Pap smear^a^	Monitoring &Evaluation, Strategic Information Department, Ministry of Health: Annual National Sexual and Reproductive Health programme report, 2015, Pages 15–16 Health Management Information System (HMIS	Private hospital or Market price [41]
**Lesions and cancer diagnosis and treatment**		
Treatment with LEEP^d^	Raleigh Fitkin Memorial (RFM) hospital-Theatre records review	Market price [41]
Treatment with cryotherapy (VIA positives)^b^	Monitoring &Evaluation Ministry of Health	Market price [41]
Follow up with Pap smear and antibiotics of LSIL	Mbabane gynaecologist (Interview)	Market price [41]
Treatment of HSIL-FIGO I with hysterectomy	Mbabane Government and Mbabane Clinic private gynaecologists (Interviews)	Private: The clinic or Market price [41]
Treatment of FIGO II-IV with radiotherapy and chemotherapy	Phalala Fund and South African private hospitals charges	Phalala and Life Hospital (Oncology unit: Radiology [42]
Biopsy^c^	Health Management Information System (HMIS)	Private hospital or Market price [41]
**Genital warts**		
Wart removal with cauterization	Theatre records review (Mbabane Government hospital, Piggs Peak hospital, RFM, Mankayane hospital, Good Shepard Hospital, Hlathikhulu Hospital). Reported by the Gynaecologists from these hospitals	Private Hospital or Market price [41]
Wart removal with podophyllotoxin and antibiotics	Mbabane gynaecologist and cancer screening clinic (Interview)	Market price [41]

^a^: The costs for Pap smear included nurse assessment, laboratory costs, physician assessment of abnormal results and test costs.

^b^: The cost for ‘see-and-treat approach included VIA testing, nurse assessment of the abnormal results, and cryotherapy treatment of the VIA positive test results.

^c^:The cost for biopsy included laboratory costs, physician assessment of abnormal results.

^d^: Costs for LEEP and TAH included physician assessment, operation cost, anaesthesia and antibiotics.

**NB:** Costs for the treatment variables were then taken from the private hospitals in Swaziland and South Africa, which were based on the market price.

### Cervical lesions

In Swaziland, the following two approaches are currently used to detect the precancerous or cancerous cells on the cervix and management of cervical lesions: (1) Pap smear and if HSIL is detected it is followed by treatment with loop electrode excision procedure (LEEP) or total adnominal hysterectomy (TAH), and (2) the “Screen-and-Treat” approach using VIA followed by treatment with cryotherapy and LEEP [43]. Data on the number of cytological/Pap results or number of women screened by Pap smear (i.e. results that could be determined, including normal or abnormal results) and inadequate cytological results (i.e. results that could not be determined) were obtained from the Annual National Sexual and Reproductive Health programme report, 2015 [44], and the Health Management Information System (HMIS) report [45]. To estimate the burden of HSIL we used survey weights to extrapolate sample proportions to population totals based on the 2007–2030 population projections aligned to the estimated 2014 population [46]. The cervical cancer screening guidelines provided information on the management of abnormal cytological results, treatment procedures for women with histologically confirmed HSIL or worse (HSIL+) [43]. As per the guidelines, it was assumed that women with abnormal cytological results were referred to a gynaecologist for an immediate biopsy and histologically confirmed CIN2+, and had one follow-up visit to undergo loop electrode excision procedure (LEEP) or total adnominal hysterectomy (TAH), which are the most common treatment currently utilized in health care facilities in Swaziland. No treatment procedure was included for women with LSIL, as the recommendations state that further follow-up should be performed every 6 months until the lesion regresses to normal or there is evidence of progression [43]. One follow-up procedure with Pap smear and antibiotics was therefore assumed to have been performed within the year of follow-up.

In 2009, the Swazi Ministry of Health integrated the “see-and-treat” approach using VIA followed by treatment with cryotherapy for the national cervical cancer prevention programme to accelerate the early detection of cervical lesions. Data on the number of VIA screened and VIA positives were obtained from Annual National Sexual and Reproductive Health programme report [44]. The total number of women treated with LEEP was retrieved from the Raleigh Fitkin Memorial (RFM) Hospital theatre records from2015.

### Cervical cancer

The resources used to calculate the treatment costs for cervical cancer were based on the Phalala fund scheme report [47], and the breakdown of the treatment variables was influenced by a Swedish study [48]. Costs for the treatment variables were then taken from the private hospital in South Africa, which were based on the market price [42]. These costs included diagnosis, staging, and full treatment of cervical cancer divided into the different cancer stages, pharmaceuticals, and transportation cost of patients from Swaziland to South Africa. Due to the lack of official data, the proportion of women treated for different International Federation of Gynecology and Obstetrics (FIGO) stages of cancer, was based on the empirical number of patients diagnosed and treated according to the cancer registry situational analysis report [49], as well as the Phalala report [47].

Direct medical costs of visits for treatment of large warts which included diathermy, cauterization, or surgical excision, were estimated at market price and based on private hospital charges. Costs for podophyllotoxin and antibiotics treatments were estimated at the market cost. To obtain the overall costs of genital warts, we used the estimated percentage of people who had related procedures or visit hospitals, multiplied by the relevant number of cases of genital warts (i.e. number of people treated with cauterization). This number was then calculated by multiplying the cost of the treatment or visit, including the cost of podophyllotion applied by a gynaecologist/nurse at the site care and one course of antibiotic.

According to the Report On Cases Of Cancers In Swaziland, a total of 440 cervical cancer cases were diagnosed between 1 January 2014 and 31^st^ December 2015 [49]. The sources of information for this report were government, private hospitals and the Hospice At Home and death certificates stating cervical cancer as the contributory cause of death. To estimate the total number of cervical cancer cases for the year 2015, we divided the 440 by 2. Therefore, for our analysis, we used the average prevalent number of 220 cervical cancer cases in Swaziland.

We assumed that the reported number of women diagnosed with cervical cancer were clinically staged according to FIGO staging procedures, and treated as per cervical cancer guidelines [43] the same year that they were diagnosed. The size of growth of the cancer is assessed clinically and supplemented by a limited of investigations (cystoscopy, cone biopsy, endocervical curettage or smear, X-ray or CT scan). Some tests for staging such as MRI are not available in the country and patients are transferred to South Africa for all treatment of invasive cervical cancer (radiotherapy, chemotherapy, and surgery), performed in South African private hospitals. Therefore, all costs for treatment were based on the private hospital charges.

### Genital warts

Due to lack of an STI surveillance system being in place, no reliable prevalence data were obtained. Therefore, epidemiological data from the current study (not yet published but under review by Infectious Agent and Cancer Journal) were used to estimate the actual population burden of genital warts in women of reproductive age (15–49 years). In the current study, we used survey weights to extrapolate sample proportions to population totals, based on the 2007–2030 population projections aligned to the estimated 2014 population (15–49 years) [46]. To calculate the cost we used the estimated population burden of genital warts obtained from our study. These estimates were deemed the best available evidence to estimate the comprehensive burden of genital warts in Swaziland, in respect of prevalent cases, persistence, and recurrence among the population. In Swaziland, genital warts are treated based on the WHO guidelines for management of STIs [50]. During data collection, gynaecologists and nurses with extensive clinical experience in managing and treating patients with genital warts were interviewed. Small genital warts are treated with podophyllotoxin and antibiotics and large warts are removed by cauterization at the hospital theatre.

### Ethical consideration

The study was approved by the Swaziland Scientific Ethics Committee (MH599C/FW00015267/IRB0009688) and the Biomedical Research Ethics Committee of the University of KwaZulu-Natal (BE 242/14).

## Results

### Cervical lesions

In 2015 the National Sexual and Reproductive Health programme and Health Management Information System reported a total of 20 009 women screened, of which 8229 (41.1%) were cytological tests (Pap smear), 11 248 (56.2%) were VIA screened and 532 (2.7%) had a biopsy [44]. Of the total screened, 16 496 (82.4%) were abnormal results. The report did not provide the number of those who were HSIL. To estimate the burden of HSIL (actual number of HSIL), we used survey weights to extrapolate sample proportions to population totals based on the 2007–2030 population projections aligned to the estimated 2014 population [46]. The collection of data on costs resulted in cost estimations for precancerous lesions and genital warts presented in Table 2.

**Table 2:** Costs for prevention, management and treatment for cervical lesions and genital warts expressed in $ 2015.

Parameter	Variables included in the cost	Cost ($)
**Pap smear screens**	Examination table with foot supports, examination light, speculum, examination gloves, cervical spatula and cytobrush glass slide and fixative, time	22
**Total VIA screened**	Nurse’s time, examination table, speculum, light source (halogen torch or flashlight), instrument tray, 3 cotton swabs, examination gloves, 20mls of 3% to 5% acetic acid (white table vinegar) solution	53
**Biopsy**	Colposcope: Variable fixed power or zoom lens (3× to 7× low power to 15× to 40× high power), biopsy forceps, endocervical curette (Kevorkian curette, no basket), endocervical speculum (Kogan, both narrow and wide types), ring forceps, tenaculum (rarely used), cervical hook (rarely used), Pap smear materials, vaginal speculums, full-strength Lugol's iodine solution, Monsel's solution (ferric subsulfate), 1 mL, Acetic acid solution 3% to 5% (white vinegar; 4–6 oz or 120–180 mL), cotton- or rayon-tipped swabs (8–10),junior Scopettes/OB-GYN applicators (6–10) 4 × 4 gauze, urine or sputum cups for vinegar, vaginal side wall retractor, underpads ("chuck pads") (17 × 24 inch), Cotton balls (15–20) Power (electricity), Dr’s time, Lab cost	58
**LEEP**	Usage of all equipment/instruments, linen and consumables: 1 packet of cotton balls, 1 packet gauze, antiseptic solutions –Savlon ±50cc, floor cleaning solution-sonic 1backet, specimen bottle, formalin 10cc, Cidex solution 5L, vinegar solution, anesthesia, doctor and nurse’s time	2 049
**Cryotherapy**	Usage of cryotherapy machine and equipment, Nitrous oxide, sanitary pad, detergents (95% alcohol, water, sodium chloride solution, Nurse’s time Antibiotics treatment: Amoxylin/ciproflaxin 500mg BID, Flagyl 400mg tid 7/7 days OR: Panado 1gm TDS and Diclofenac 50mg TDS 3/7 days	96
**Follow up procedure of LSIL**	With Pap smear and antibiotics.	52
**Treatment of HSIL**	With hysterectomy during anesthesia: Usage of equipment/instruments, linen, 5 swabs abdominal packs, 20 gauges, Sutures –4 vicryl No.2 and 2 vicryl No.1, anesthesia, doctor and nurse	3 941
**Wart removal with cauterization**	Usage of diathermy machine/instruments, linen, antibiotics, 5 abdominal swaps, sutures	2 948
**Wart removal with topical cream**	Silver nitrate pencil, gauze, podophyllotoxin, antibiotics, nurse’s time	161

### Cervical cancer

Table 3 shows the breakdown for the staging and treatment variables for FIGO 1–4 (total number of sessions per stage). According to the Swaziland National Cancer Registry (SNCR), there were 220 number cervical cancer cases during 2015 in the country [49]. The treatment period was assumed to occur within the same 1 year as the staging. Staging included a clinical examination with cystoscopy during anaesthesia, performed in South Africa. FIGO stages 1a1-1b1 were assumed to be treated with surgery alone (i.e. hysterectomy) in Swaziland and no transport or lodging was therefore estimated. In FIGO stage 1b2-5, the patients were assumed to be treated with brachytherapy, radiotherapy and chemotherapy during the 5 weeks and therefore lodging was assumed for 35 days. The difference in treatment between FIGO stage 3 and FIGO stage 4 was due to the fact that, the dissemination of disease in stage 4 needed limited brachytherapy.

**Table 3:** Breakdown for staging and treatment variables for FIGO stage 1–4.

Variables	1a1-1b1	1b2-2	3	4
Transport		1	1	1
Lodging		35	35	35
Hospital admission	1	1	1	1
Total adnominal hysterectomy with anaesthesia	1	-	-	-
Clinical examination with cystoscopy with anaesthesia	1	1	1	1
Brachytherapy treatment	-	3	2	-
Radiotherapy treatment	-	25	25	25
Follow up during treatment	-	2	2	2
Chemotherapy	-	6	6	6
Follow-up during chemotherapy	-	1	1	1
Follow-up of treatment	1	12	12	12
MRI	1	1	1	1

Table 4 shows the costs for staging, management and treatment of cervical cancer per FIGO stages. Including costs in the breakdown variables resulted in a cost of $6 405 for FIGO 1a1-1b1 and between $25 527 and $23 923 for FIGO 1b2-4. Less costly was FIGO stage 4 due to less brachytherapy.

**Table 4:** Costs for staging, management and treatment of cervical cancer per FIGO stage 1–4 presented in USD ($) 2015.

Staging and treatment variables	Cost ($)	1a1-1b1	1b2-2	3	4
Transport	236		236	236	236
Lodging	22		765	765	765
Hospital admission	591	591	591	591	591
Total adnominal hysterectomy with anaesthesia	5 517	5 517			
Clinical examination with cystoscopy with anaesthesia	660		660	660	660
Brachytherapy treatment	535		1 605	1 070	
Radiotherapy treatment	535		13 371	13 371	13 371
Follow-up during brachytherapy and radiotherapy	296		593	593	593
Chemotherapy	514		3 083	3 083	3 083
Follow-up during chemotherapy	296		296	296	296
Follow-up after and during treatment	296	296	3 557	3 557	3 557
MRI	771		771	771	771
**Total**		**6 405**	**25 527**	**24 992**	**23 923**

The total annual cost for cervical cancer was estimated at $5 million (ranging from $3.8 million to $6.3 million) based on estimates of 10% of patients in FIGO stage Ia1-Ib1, 10% in FIGO stage Ib2-II, 50% in FIGO stage 3 and 30% in FIGO stage 4 (Table 5).

**Table 5:** Annual cost estimation for prevention, management and treatment of cervical lesions, cancer and genital warts expressed in $ 2015.

Parameter	Prevalence 2015	Cost per item	Base case cost	Range	
	Number	$ 2015	$ 2015	Lower (-50%)	Higher (+50)
**Screening**					
Pap smear	8 229	22	180 301	90 150	270 451
Total VIA screened	11 248	53	601 050	300 525	901 576
Biopsy	532	158	83 859	41 929	125 788
	**20 009**		**865 210**	**432 605**	**1 297 815**
**Diagnosed cervical lesions**					
Treatment with LEEP	309	2 049	633 195	316 598	949 793
VIA positive treated with cryotherapy	1 408	96	169 647	84 823	254 470
Follow up of LSIL with Pap smear 6 months later and antibiotics (3 drugs)	12 862	52	664 995	332 497	997 492
Treatment of HSIL with hysterectomy during anesthesia	1 917	3 941	7 554 366	3 777 183	11 331 549
	**16 496**		**9 022 203**	**4 511 102**	**13 533 305**
**Diagnosed with cervical cancer**					
FIGO I-Ib1	22	6 405	140 899	70 450	211 349
FIGO Ib2-II	22	25 527	561 601	280 800	842 401
FIGO III	110	24 992	2 749 172	1 374 586	4 123 759
FIGO IV	66	23 923	1 578 905	789 453	2 368 358
	**220**		**5 030 578**	**2 515 289**	**7 545 867**
**Genital warts**					
Wart removal with cauterization	116	552	63 997	31 999	95 996
Wart removal with Podophyllotoxin and antibiotics	5 995	161	966 720	483 360	1 450 080
	**6 111**		**1 030 717**	**515 359**	**1 546 076**
**Total**	**42 836**		**15 948 708**	**7 974 354**	**23 923 062**

### Genital warts

The total number of prevalent cases of genital warts (seeking or receiving treatment) in Swaziland was estimated to be 6 111 in 2015 (Table 5). The estimated costs for management and treatment of genital warts were almost $1.1 million of which topical medicines represented the majority of the $0.9 million. Treatment with cauterization represented only a minor cost.

### Total annual estimated cost

The estimated total annual cost of prevention and health care with cervical screening was $0.9 million (ranging between $0.6 million and $1.1million), while management and treatment of cervical lesions was estimated at $9 million (ranging between $6.7 million and $11.3 million), of which costs for treatment with radical hysterectomy represented the largest cost of $7.6 million in the base case estimate (Table 5).

The total direct medical cost for the cervical screens, management and treatment of cervical lesions, cervical cancer and genital warts in 2015 was estimated at $16 million in the base case. To investigate the impact on the result we varied the input variables by ±50% and the total annual cost estimate ranged between $8 and $24 million. To reflect the number of unrecorded cases in the public or private hospitals, we increased the prevalence rates by 25% and the costs were then estimated at $19.9 million (ranged between $10 million and $30 million).

## Discussion

This study is the first to estimate the economic burden including the direct medical costs of HPV-related diseases including precancerous lesions, cervical cancer and genital warts in Swaziland. The largest cost in the analysis-cost was for treatment of high-grade lesions $7.6 million (ranging between $4.5and $13.5 million) followed by costs for treatment of cervical cancer estimated at $5 million (ranging between $2.5 and $7.5 million). Management of anogenital warts was estimated at $1 million (ranging between $0.5 million and $1.5 million). The cervical HPV-related conditions incur a major economic burden worldwide [51]. In countries where there is organized screening, the major cost represents prevention whilst in countries that lack screening, costs for management and treatment are larger. In this study, the large cost is estimated for treatment of high-grade lesions and treatment of cervical cancer both requiring extensive treatments at high costs and is therefore representing 80% of the total costs. Screening alone only represents a minor cost with 5% of the total costs. Both clinical conditions are mainly detected when patients seek medical care for symptoms. Organized screening programs with high population coverage would lead to earlier detection of cervical dysplasia and cancer and following costs for treatment would then be much lower and a higher percentage of the total cost would be estimated for screening. However, an organized screening program is today lacking in Swaziland. We did not estimate cost for the non-cervical HPV-related cancers, which also affect males and would increase the total estimated costs significantly.

In the United States with organized screening, cervical cancer and cervical intraepithelial neoplasia (CIN) are the most costly HPV-related conditions, and are estimated to account for the direct medical cost of $4.6 billion annually [52]. When the cost of managing anogenital warts and other oncogenic HPV-associated cancers are included, the total costs rise to $5 billion per year [52]. According to another study, an increase in the direct medical cost associated with HPV-related conditions has been noted in the United States; in 2010, the overall annual direct medical cost of preventing and treating HPV-associated diseases was estimated at $8 billion [53]. Out of this total cost, the majority of $6.6 billion (82%) represented routine cervical cancer screening and follow-up, whilst $1 billion (12%) was for cancer (including $0.4 billion for cervical cancer and $0.3 billion for oropharyngeal cancer); $0.3 billion (3.6%) was for anogenital warts and $0.2 billion for recurrent respiratory papilomatosis (RRP) [53]. A study done in Belgium found that the annual direct medical costs of cervical cancer, cervical dysplasia, and genital warts combined were estimated at approximately $12 million, with around 60% and 20% of the costs for cervical cancer and CIN management, respectively [54]. In Germany, the total annual cost of management of genital warts was estimated at $52.3 million from a third-party payer perspective (indirect cost excluded) and $57.7 million from the societal perspective (indirect costs included), corresponding to an average cost per patient of $570 and $648, respectively [55]. In France, the costs for HPV-related cancers were estimated according to the percentage of each cancer type attributable to HPV infection. The total costs were estimated to $253.6 million [56]. The overall costs in men were $114.3 million, driven mainly by head and neck cancers ($100.9 million) and in women were $141.4 million, due mainly to invasive cervical cancer ($89.5 million) [56]. In Italy, the total direct costs associated with the annual incident cases of the nine HPV-related conditions included in the analysis were estimated to be $564 million [57]. Furthermore, the fraction attributable to HPV 6, 11, 16, and 18 was $310.5 million, accounting for approximately 55% of the total annual burden of HPV-related disease [57]. Another study performed from a societal perspective in Sweden estimated costs in 2009 for cervical lesions, cervical cancer and genital warts at $113.7 million, of which $868 million (76%) were direct medical costs and the majority of the cost for the organized screening programme [40]. The described studies above demonstrate the diversity of how the costs are distributed between prevention and treatment as well as between direct and indirect costs. Guidelines for economic studies differ between countries and costs are presented differently. However, there are more cost-of-illness studies investigating the costs for HPV related diseases estimated in western countries than in African countries. This study is therefore unique and useful to health policy makers for planning and budget decisions within Swaziland. It is important to foresee the future HPV prevalence infection and related carcinogenesis among the population to plan for increase in costs for treatment and prevention of HPV related diseases.

In low-resource countries with a lack of organized screening such as Swaziland, the HPV vaccine is an effective alternative prevention strategy and may be associated with less direct medical costs annually than screening and management at the local health care site [58]. The HPV vaccine has proved a robust immune response to the HPV virus and due to the lack of organized screening in Swaziland should be used for the prevention of cervical cancer. Coverages above 50% in HPV immunization programs among either young females or males are suggested to give herd immunity [59]. It is proven that anogenital warts have decreased among both the vaccinated female population and the unvaccinated male population in countries that have introduced organized HPV vaccination [60, 61]. To estimate the costs related to HPV vaccination in Swaziland we used the direct medical costs for the HPV vaccine per dose for HPV-2 and HPV-4 being $120 and HPV-9 $135 in 2016 prices, respectively, based on the US Centers for Disease Control and Prevention public sector cost [62]. We then extrapolated the 2007–2030 population projections aligned to the 2016 population estimates for the total of boys and girls aged 9–12 years to estimate the population size. We then multiplied costs with population size to estimate the vaccine cost in different population scenarios.

If current national young females and males in Swaziland between 9-12-year-olds should be offered immunization against HPV with the current recommendation of a 2 dose schedule for HPV-2, HPV-4 and 3 doses for HPV-9, the estimated direct medical costs would be a one-time cost of approximately $24 million or $40.6 million with HPV-2, HPV-4 or HPV-9 respectively, following an annual cost estimation including only one birth cohort of females plus males, of approximately $5.5 million or $9.2 million (with HPV-2, HPV-4 or HPV-9 respectively, offered to 9-year-olds), annually based on the number of individuals in each year group (Table 6). Only including pre-adolescent females would result into a cost estimate of $ 11.8 million and 20 million with HPV-2,HPV-4 and HPV-9 respectively, for all 9-12-year-olds females and an annual cost of $2.7 and $4.6 million respectively (9-year-old girls). Logistics and administration of the vaccine would be an added cost to this estimate.

**Table 6:** The 2007–2030 population projections aligned to the 2016 population (estimates total of boys and girls aged 9–12 years) and vaccine costs presented in $ 2015.

Ages	Boys	Girls	Total	HPV-2, 4 cost ($)*	HPV-9 cost ($)**
9	11 518	11 242	22 760	5 462 400	9 217 800
10	12 825	12 328	25 153	6 036 720	10 186 965
11	13 244	12 791	26 035	6 248 400	10 544 175
12	13 283	12 962	26 245	6 298 800	10 629 225
**Total**	**50 870**	**49 323**	**100 193**	**24 046 320**	**40 578 165**

*Cost estimation with 2 doses schedule as current recommendations for HPV-2, 4.

** Cost estimation with 3 doses schedule as current recommendation for HPV-9.

**Source**: Swaziland population projections 2007–2030 [46]

Since oncogenic HPV types also cause a several significant diseases in both females and males, namely cancer of the vulva, vagina, anus, penis and oropharynx [63] public investment in vaccination of young females and males is therefore suggested to decrease the public health care costs and human burden in the next generations. A study from Australia showed a significant decrease in the prevalence of vaccine-targeted HPV types, possible cross-protective effects in the same vaccinated group and herd immunity among the unvaccinated females [64]. Another study from Scotland observed a similar reduction in HPV in the unvaccinated population, but the effect of herd immunity is difficult to fully establish due to a continuous smaller number of unvaccinated females in countries with national immunization programmes [65]. To exclude males from HPV immunization programmes, a coverage of 80% is suggested to establish herd immunity among the unvaccinated population [66]. The reduction in the prevalence of HPV infection should reduce the number of cervical lesions, cervical cancer and genital warts over time, with subsequently reduced needs in management and treatment, and the future reduction in the human and economic burden of HPV-related diseases and related deaths due to HPV-related cancers.

Even though costs presented in this study are substantial, it is likely to be a major underestimation of actual costs. Important limitations of this study are that it only included costs related to the direct medical health care costs and excluded the costs for the patients. In addition, palliative care occurring outside a hospital setting, or other costs like patient transportation were also excluded. Informal care from family members was also excluded and this is expected to impact on the cost estimations. Indirect costs (i.e., lost productivity due to illness) were also excluded, which could lead to an underestimation of the societal benefits in making decision about public interventions such as HPV immunization. Costs for adverse outcomes such as preterm births associated with treatment of cervical lesions and decreased quality of life associated with the cervical HPV-related diseases, were excluded in this study. A further economic analysis should therefore include the societal costs (including both direct and indirect costs) for all known HPV-related illnesses to present the total cost of HPV-related diseases for both sexes. Such as estimate would then reflect the total HPV-related economic burden for the society in countries that have implemented an HPV immunization program, there is an expected decrease in HPV-related conditions in future generations. To guide public health policy in Swaziland, a cost-effectiveness analysis (CEA) projecting both health and economic outcomes in “real life scenarios”, should be performed. The CEA should include all costs from HPV-related conditions and benefits from HPV vaccination for both sexes to evaluate the allocation of resources for a national HPV immunization program among the pre-adolescents.

## Conclusions

The economic burden of HPV-related cervical diseases and genital warts represents a major public health issue in Swaziland. Prevention of HPV infection with a national HPV immunization program for pre-adolescents would prevent the majority of HPV-related conditions and the related deaths. Large cost savings would then result for public health care resources utilized for the management and treatment of cervical lesions, cervical cancer and genital warts in Swaziland.

## References

[1] Shavit O, Raz R, Stein M, Chodick G, Schejter E, Ben-David Y, et al Evaluating the epidemiology and morbidity burden associated with human papillomavirus in Israel: accounting for CIN1 and genital warts in addition to CIN2/3 and cervical cancer. Appl Health Econ Health Policy. 2012;10(2):87–97. Epub 2011/12/29. 10.2165/11594780-000000000-00000 . 22201263

[2] Mayeaux EJ Jr. Reducing the economic burden of HPV-related diseases. J Am Osteopath Assoc. 2008;108(4 Suppl 2):S2–7. Epub 2008/05/29. . 18463361

[3] Elfstrom KM, Herweijer E, Sundstrom K, Arnheim-Dahlstrom L. Current cervical cancer prevention strategies including cervical screening and prophylactic human papillomavirus vaccination: a review. Curr Opin Oncol. 2013 Epub 2013/11/20. 10.1097/cco.0000000000000034 . 24248011

[4] Armstrong EP. Prophylaxis of cervical cancer and related cervical disease: a review of the cost-effectiveness of vaccination against oncogenic HPV types. J Manag Care Pharm. 2010;16(3):217–30. Epub 2010/03/25. 10.18553/jmcp.2010.16.3.217 20331326 PMC10437588

[5] Merck & Co. Highlights of prescribing information. Gardasil (human papillomavirus quadrivalent [types 6, 11, 16, and 18] vaccine, recombinant] suspension for intramuscular injection Revised October 2009.

[6] GlaxoSmithKline. Highlights of prescribing information. Cervarix (human papillomavirus bivalent [types 16 and 18] vaccine, recombinant]) suspension for intramuscular injection. Revised October 2009.

[7] David MP, Van Herck K, Hardt K, Tibaldi F, Dubin G, Descamps D, et al Long-term persistence of anti-HPV-16 and -18 antibodies induced by vaccination with the AS04-adjuvanted cervical cancer vaccine: modeling of sustained antibody responses. Gynecol Oncol. 2009;115(3 Suppl):S1–6. Epub 2009/02/17. 10.1016/j.ygyno.2009.01.011 . 19217149

[8] Schwarz TF, Spaczynski M, Schneider A, Wysocki J, Galaj A, Perona P, et al Immunogenicity and tolerability of an HPV-16/18 AS04-adjuvanted prophylactic cervical cancer vaccine in women aged 15–55 years. Vaccine. 2009;27(4):581–7. Epub 2008/11/22. 10.1016/j.vaccine.2008.10.088 . 19022320

[9] Harper DM, Paavonen J. Age for HPV vaccination. Vaccine. 2008;26 Suppl 1:A7–11. Epub 2008/07/22. . 18642467 10.1016/j.vaccine.2008.01.013

[10] Harper DM. Impact of vaccination with Cervarix (trade mark) on subsequent HPV-16/18 infection and cervical disease in women 15–25 years of age. Gynecol Oncol. 2008;110(3 Suppl 1):S11–7. Epub 2008/07/25. 10.1016/j.ygyno.2008.06.029 . 18649932

[11] Weisberg E, Bateson D, McCaffery K, Skinner SR. HPV vaccination catch up program—utilisation by young Australian women. Aust Fam Physician. 2009;38(1–2):72–6. Epub 2009/03/14. . 19283241

[12] Joura EA, Giuliano AR, Iversen OE, Bouchard C, Mao C, Mehlsen J, et al A 9-valent HPV vaccine against infection and intraepithelial neoplasia in women. The New England journal of medicine. 2015;372(8):711–23. Epub 2015/02/19. 10.1056/NEJMoa1405044 . 25693011

[13] Petrosky E, Bocchini JA Jr., Hariri S, Chesson H, Curtis CR, Saraiya M, et al Use of 9-valent human papillomavirus (HPV) vaccine: updated HPV vaccination recommendations of the advisory committee on immunization practices. MMWR Morbidity and mortality weekly report. 2015;64(11):300–4. Epub 2015/03/27. . 25811679 PMC4584883

[14] Harper DM, Williams KB. Prophylactic HPV vaccines: current knowledge of impact on gynecologic premalignancies. Discov Med. 2010;10(50):7–17. Epub 2010/07/31. . 20670593

[15] Shi JF, Chen JF, Canfell K, Feng XX, Ma JF, Zhang YZ, et al Estimation of the costs of cervical cancer screening, diagnosis and treatment in rural Shanxi Province, China: a micro-costing study. BMC Health Serv Res. 2012;12:123 Epub 2012/05/26. 10.1186/1472-6963-12-123 ; 22624619 PMC3461448

[16] Levin CE, Sellors J, Shi JF, Ma L, Qiao YL, Ortendahl J, et al Cost-effectiveness analysis of cervical cancer prevention based on a rapid human papillomavirus screening test in a high-risk region of China. International journal of cancer. 2010;127(6):1404–11. Epub 2010/01/06. 10.1002/ijc.25150 . 20049838

[17] Goldie SJ, Diaz M, Kim SY, Levin CE, Van Minh H, Kim JJ. Mathematical models of cervical cancer prevention in the Asia Pacific region. Vaccine. 2008;26 Suppl 12:M17–29. Epub 2008/11/01. 10.1016/j.vaccine.2008.06.018 . 18945411

[18] Goldie SJ, Gaffikin L, Goldhaber-Fiebert JD, Gordillo-Tobar A, Levin C, Mahe C, et al Cost-effectiveness of cervical-cancer screening in five developing countries. The New England journal of medicine. 2005;353(20):2158–68. Epub 2005/11/18. 10.1056/NEJMsa044278 . 16291985

[19] Mandelblatt JS, Lawrence WF, Gaffikin L, Limpahayom KK, Lumbiganon P, Warakamin S, et al Costs and benefits of different strategies to screen for cervical cancer in less-developed countries. J Natl Cancer Inst. 2002;94(19):1469–83. Epub 2002/10/03. . 12359856 10.1093/jnci/94.19.1469

[20] Goldhaber-Fiebert JD, Goldie SJ. Estimating the cost of cervical cancer screening in five developing countries. Cost Eff Resour Alloc. 2006;4:13 Epub 2006/08/05. 10.1186/1478-7547-4-13 ; 16887041 PMC1570139

[21] Legood R, Gray AM, Mahe C, Wolstenholme J, Jayant K, Nene BM, et al Screening for cervical cancer in India: How much will it cost? A trial based analysis of the cost per case detected. International journal of cancer. 2005;117(6):981–7. Epub 2005/07/09. 10.1002/ijc.21220 . 16003735

[22] Quentin W, Adu-Sarkodie Y, Terris-Prestholt F, Legood R, Opoku BK, Mayaud P. Costs of cervical cancer screening and treatment using visual inspection with acetic acid (VIA) and cryotherapy in Ghana: the importance of scale. Trop Med Int Health. 2011;16(3):379–89. Epub 2011/01/11. 10.1111/j.1365-3156.2010.02722.x ; 21214692 PMC3429861

[23] Hu D, Goldie S. The economic burden of noncervical human papillomavirus disease in the United States. Am J Obstet Gynecol. 2008;198(5):500 e1–7. Epub 2008/05/06. 10.1016/j.ajog.2008.03.064 ; 18455524 PMC2441758

[24] World Health Organization. World health report. Exceutive summary. Health of adults. WHO, 2016.

[25] Barnighausen T, Bloom DE, Cafiero ET, O'Brien JC. Economic evaluation of vaccination: capturing the full benefits, with an application to human papillomavirus. Clin Microbiol Infect. 2012;18 Suppl 5:70–6. Epub 2012/08/14. 10.1111/j.1469-0691.2012.03977.x . 22882176

[26] Cutts FT, Franceschi S, Goldie S, Castellsague X, de Sanjose S, Garnett G, et al Human papillomavirus and HPV vaccines: a review. Bull World Health Organ. 2007;85(9):719–26. Epub 2007/11/21. ; 10.2471/BLT.06.038414 18026629 PMC2636411

[27] Lacey CJ, Lowndes CM, Shah KV. Chapter 4: Burden and management of non-cancerous HPV-related conditions: HPV-6/11 disease. Vaccine. 2006;24 Suppl 3:S3/35–41. Epub 2006/09/05. 10.1016/j.vaccine.2006.06.015 . 16950016

[28] Campos NG, Kim JJ, Castle PE, Ortendahl JD, O'Shea M, Diaz M, et al Health and economic impact of HPV 16/18 vaccination and cervical cancer screening in Eastern Africa. International journal of cancer. 2012;130(11):2672–84. Epub 2011/07/01. 10.1002/ijc.26269 ; 21717458 PMC3314721

[29] Vijayaraghavan A, Efrusy M, Lindeque G, Dreyer G, Santas C. Cost effectiveness of high-risk HPV DNA testing for cervical cancer screening in South Africa. Gynecol Oncol. 2009;112(2):377–83. Epub 2008/12/17. 10.1016/j.ygyno.2008.08.030 . 19081611

[30] Insinga RP, Dasbach EJ, Elbasha EH. Assessing the annual economic burden of preventing and treating anogenital human papillomavirus-related disease in the US: analytic framework and review of the literature. Pharmacoeconomics. 2005;23(11):1107–22. Epub 2005/11/10. . 16277547 10.2165/00019053-200523110-00004

[31] Drummond O B. S GL, Torrance G.W. Methods for the evaluation of health care programmes. 3rd ed Oxford: Oxford Medical Publications; 2005.

[32] United Nations Environment Programme (UNEP)/GRID. Topographic Maps: Southern Africa,topographic and political maps Geneva2005 [cited 2013 10/02/20013]. http://www.grida.no/graphicslib/detail/southern-africa-topographic-and-political-map_1515.

[33] Macmillan. Geography and map of Swaziland Matsapha 2005 [cited 2012 13/12/2012]. http://geography.about.com/library/cia/blcswaziland.htm.

[34] Central Statistic office. Sawziland Population and Housing Census. Mbabane, Swaziland: Central Statistic office, 2007.

[35] Centers for Disease Control and Prevention. Centers for Disease Control and Prevention. CDC vaccine price list. Atlanta: 2015 14/03/2015. Report No.

[36] TrADING Economics. Swaziland GDP per Capita [Internet]. 2016 [cited 30/03/2017]. http://www.tradingeconomics.com/swaziland/gdp-per-capita.

[37] The Joint United Nations Programme on HIV/AIDS (UNAIDS). Swaziland country report on monitoring the political declaration on HIV and AIDS. Mbabane: NERCHA, 2012.

[38] Ginindza TG, Dlamini X, Almonte M, Herrero R, Jolly PE, Tsoka-Gwegweni JM, et al Prevalence of and Associated Risk Factors for High Risk Human Papillomavirus among Sexually Active Women, Swaziland. PloS one. 2017;12(1):e0170189 Epub 2017/01/24. 10.1371/journal.pone.0170189 ;. 28114325 PMC5256897

[39] Rice DP, Hodgson TA, Kopstein AN. The economic costs of illness: a replication and update. Health care financing review. 1985;7(1):61–80. Epub 1986/03/06. ; 10311399 PMC4191510

[40] Ostensson E, Froberg M, Leval A, Hellstrom AC, Backlund M, Zethraeus N, et al Cost of Preventing, Managing, and Treating Human Papillomavirus (HPV)-Related Diseases in Sweden before the Introduction of Quadrivalent HPV Vaccination. PloS one. 2015;10(9):e0139062 Epub 2015/09/24. 10.1371/journal.pone.0139062 ; 26398189 PMC4580320

[41] The Clinic Group. Private Health Care Services Cost/Pricing 2015. Mbabane, Swaziland.

[42] Hopelands Cancer Centre. Cervical Cancer Treatment Cost. Pietermaritzburg, South Africa 2016.

[43] The Kingdom of Swaziland. Cervical Cancer Screening Guidelines. Mbabane: Ministry of Health, 2013.

[44] The Kingdom of Swaziland. Annual National Sexual and Reproductive Health Programme Report. Ministry of Health, Monitoring and Evaluation Unit SID; 2015.

[45] The Kingdom of Swaziland. Health Management Information System (HMIS) Report. 2015.

[46] The Kingdom of Swaziland Gorvernment and UNFPA. Swaziland Population Projections 2007–2030, Central Statistics Office (CSO), editor. 2007.

[47] Phalala Fund Administration Office. Annual Report year 2014/15. Mbabane,Swaziland: 2015.

[48] Ostensson E, Hellstrom AC, Hellman K, Gustavsson I, Gyllensten U, Wilander E, et al Projected cost-effectiveness of repeat high-risk human papillomavirus testing using self-collected vaginal samples in the Swedish cervical cancer screening program. Acta obstetricia et gynecologica Scandinavica. 2013;92(7):830–40. Epub 2013/03/28. 10.1111/aogs.12143 . 23530870

[49] The Kingdom of Swaziland. Swaziland National Cancer Registry. Report on Cases of cancers in Swaziland -2014-2015. Mbabane: Ministry of Health, 2016.

[50] World Health Organization. Guidelines for the management of sexually transmitted infections. Geniva, Switzerland 2003.

[51] Low JJ, Ko Y, Ilancheran A, Zhang XH, Singhal PK, Tay SK. Health and economic burden of HPV-related diseases in Singapore. Asian Pac J Cancer Prev. 2012;13(1):305–8. Epub 2012/04/17. . 22502690 10.7314/apjcp.2012.13.1.305

[52] Lipsy RJ. Assessing the short-term and long-term burden of illness in cervical cancer. Am J Manag Care. 2008;14(6 Suppl 1):S177–84. Epub 2008/07/18. . 18611085

[53] Chesson HW, Ekwueme DU, Saraiya M, Watson M, Lowy DR, Markowitz LE. Estimates of the annual direct medical costs of the prevention and treatment of disease associated with human papillomavirus in the United States. Vaccine. 2012;30(42):6016–9. Epub 2012/08/08. 10.1016/j.vaccine.2012.07.056 . 22867718 PMC6629018

[54] Annemans L, Remy V, Lamure E, Spaepen E, Lamotte M, Muchada JP, et al Economic burden associated with the management of cervical cancer, cervical dysplasia and genital warts in Belgium. J Med Econ. 2008;11(1):135–50. Epub 2008/01/01. 10.3111/13696990801961611 . 19450115

[55] Hillemanns P, Breugelmans JG, Gieseking F, Benard S, Lamure E, Littlewood KJ, et al Estimation of the incidence of genital warts and the cost of illness in Germany: a cross-sectional study. BMC Infect Dis. 2008;8:76 Epub 2008/06/04. 10.1186/1471-2334-8-76 ; 18518976 PMC2438362

[56] Borget I, Abramowitz L, Mathevet P. Economic burden of HPV-related cancers in France. Vaccine. 2011;29(32):5245–9. Epub 2011/05/28. 10.1016/j.vaccine.2011.05.018 . 21616117

[57] Baio G, Capone A, Marcellusi A, Mennini FS, Favato G. Economic burden of human papillomavirus-related diseases in Italy. PloS one. 2012;7(11):e49699 Epub 2012/11/28. 10.1371/journal.pone.0049699 ; 23185412 PMC3504125

[58] Jit M, Brisson M, Portnoy A, Hutubessy R. Cost-effectiveness of female human papillomavirus vaccination in 179 countries: a PRIME modelling study. The Lancet Global health. 2014;2(7):e406–14. Epub 2014/08/12. 10.1016/S2214-109X(14)70237-2 . 25103394

[59] Drolet M, Bénard É, Boily MC, Ali H, Baandrup L, Bauer H, et al Population-level impact and herd effects following human papillomavirus vaccination programmes: a systematic review and meta-analysis. Lancet Infect Dis. 2015;15(5):565–80. 10.1016/S1473-3099(14)71073-4 . 25744474 PMC5144106

[60] Herweijer E, Leval A, Ploner A, Eloranta S, Simard JF, Dillner J, et al Association of varying number of doses of quadrivalent human papillomavirus vaccine with incidence of condyloma. Jama. 2014;311(6):597–603. Epub 2014/02/13. 10.1001/jama.2014.95 . 24519299

[61] Herweijer E, Sundstrom K, Ploner A, Uhnoo I, Sparen P, Arnheim-Dahlstrom L. Quadrivalent HPV vaccine effectiveness against high-grade cervical lesions by age at vaccination: A population-based study. International journal of cancer. 2016;138(12):2867–74. Epub 2016/02/10. 10.1002/ijc.30035 ; 26856527 PMC5069657

[62] Centers for Disease Control and Prevention. CDC vaccine price list. [March 14, 2015]. http://www.cdc.gov/vaccines/programs/vfc/awardees/vaccine-management/price-list/.

[63] Wiley D, Masongsong E. Human papillomavirus: the burden of infection. Obstetrical & gynecological survey. 2006;61(6 Suppl 1):S3–14. Epub 2006/05/30. 10.1097/01.ogx.0000221010.82943.8c . 16729902

[64] Tabrizi Sepehr N JM L B, Kaldor John M, et al,. Assessment of herd immunity and cross-protection after a human papillomavirus vaccination programme in Australia: a repeat cross-sectional study. Lancet Infect Dis 2014;14: 958–66. 10.1016/S1473-3099(14)70841-2 25107680

[65] Cameron RL, Kavanagh K, Pan J, Love J, Cuschieri K, Robertson C, et al Human Papillomavirus Prevalence and Herd Immunity after Introduction of Vaccination Program, Scotland, 2009–2013. Emerging Infectious Diseases. 2016;22(1):56–64. 10.3201/eid2201.150736 26692336 PMC4696690

[66] World Health Organisation. Evidence based recommendations on Human Papilloma Virus (HPV) Vaccines Schedules Background paper for SAGE discussions Geneva2014.

